# Electrochemical micro-machining based on double feedback circuits

**DOI:** 10.1038/s41598-022-25964-y

**Published:** 2023-01-06

**Authors:** Lizhong Xu, Jipeng Wang, Chuanjun Zhao

**Affiliations:** Mechanical Engineering School, Yanshan University, Qinhuangdao, 066004 China

**Keywords:** Chemical engineering, Mechanical engineering

## Abstract

Pulsed electrochemical micromachining accuracy predominantly depends on pulse duration. To obtain high accuracy, an expensive power source with ultra-short pulse duration is needed. An electrochemical micromachining method based on double feedback circuits is proposed in this work to achieve this aim. A positive feedback circuit plus a negative feedback circuit is used in the circuit of the pulsed electrochemical micromachining. Thus, the gains of the feedback circuits can control the pulse duration of the machining system. Experiments show that the machining resolution can be improved notably by an increase in the feedback gains. Using the method, one micro double cure beam is produced, and its accuracy gets to nanometer level under the condition of using an ordinary pulse duration power source.

## Introduction

Micro-machining technology is an emerging processing technology of micro-electromechanical system, which belongs to the research hot-spot in the field of advanced manufacturing technology in the world. It is of great significance to ensure and improve the working performance of micro-electromechanical system^1–3^. Among these micro machining technologies, electrochemical machining method has great development potential for processing materials on micrometer and nanometer scales because material transfer is carried out at ion scale during electrochemical machining. So, the electrochemical micro machining has attracted the attention of researchers all over the world.

Bhttacharyya developed a micro electrochemical machining method which included a machining system, electrical system, electrolyte flow system, and monitoring system, and then analyzed the possibility of its use in micro-structural machining^4^. Zhu proposed a dual pole tool with metal bushing on the outside of the insulating coating of the cathode tool, which reduced the current density in the area of the side gap of the machining hole and improved machining accuracy and stability^5^. Mount analyzed the current transient in electrochemical machining and determined the key parameters related to the machining process of the planar work-piece^6^.

Although electrochemical machining has advantages in principle, the localization of traditional electrochemical machining technology is very poor. This greatly limits the machining capability of this technology for micro and nanostructures. To increase localization of the electrochemical machining, Schuster introduced an electrochemical micromachining (ECMM) technique based on ultra-short pulse voltages, and extensive studies were carried out using the technique^7^. Shin optimized pulse signal parameters such as the voltage peak, pulse duration, and its period, to improve machining accuracy and stability^8^. Maurer fabricated microstructures on NiTi shape memory alloys using the ECMM technique based on ultrashort pulses^9^. Huang developed a nanosecond pulsed electrochemical micromachining system, conducted a series of experiments, studied the effects of pulse signal parameters on machining accuracy, and successfully prepared complex microstructures on nickel and superalloy plates^10^. Wang used a spherical tool electrode to reduce the taper of the microstructures processed^11^. Chen investigated the relationship between the machining accuracy of the technique and the pulse waveform^12,13^. Koyano machined micro-textured pattern by means of the ultra-short pulse electrochemical machining technique^14^. Zhang optimized the tool cathode structure and machining parameters in the pulsed electrochemical machining technology, and improved the machining quality of the small ring groove on the inner wall of the nozzle^15^.

In the above-mentioned ECMM technique, the machining accuracy mainly depends on the voltage pulse duration. To achieve machining accuracy of nanometer scale, picosecond scale of the pulse duration voltage is needed, which is not easy to obtain and is expensive.

For it, Xu used adjustable inductance and Zhao used adjustable capacitance in the circuit of the pulsed electrochemical micromachining so that the machining accuracy was increased by tuning the inductance or the capacitance when the power source of μs pulse width was used^16,17^.

Among these studies, machining precision based on variable capacitance or variable inductance techniques reached up to nanometer level. However, the problem in variable capacitance machining technology is that the influence of stray capacitance must be strictly controlled. In variable inductance machining technology, the peak voltage of the pulse signal depends on the inductance and should be tuned and controlled.

Feedback control is a classical electrical technique which is widely used in fields such as electromechanical systems, rotor rotation, energy systems, piezoelectric-driven mechanisms, and quantum systems, etc.

The machining circuit of the pulsed electrochemical micromachining is equivalent to an RC circuit whose time constant can be changed by adding a feedback circuit, which could alter the machining accuracy of pulsed electrochemical micromachining. Xu proposed an electrochemical micromachining method based on time constant control and investigated the relationship between machining accuracy and feedback gain of the control loop^18^.

The above processing technology still needs to be further improved to increase machining accuracy of the micro structures. For this reason, an electrochemical micromachining technique based on the double feedback principle is proposed. In addition to pulse width, two precision control parameters are provided. Compared with the methods mentioned above, the proposed technique is more advantageous for increasing machining accuracy.

## Results

### Effects of positive feedback loop gain on machining accuracy

From Eqs. (4) and (5) (see method section), it is known that increasing the positive feedback coefficient *k*_1_ can extend the time constant τ_v_ to improve machining accuracy. The verification was carried out through micro-hole machining experiments, and each group of experiments was repeated three times.

**Figure 1:**
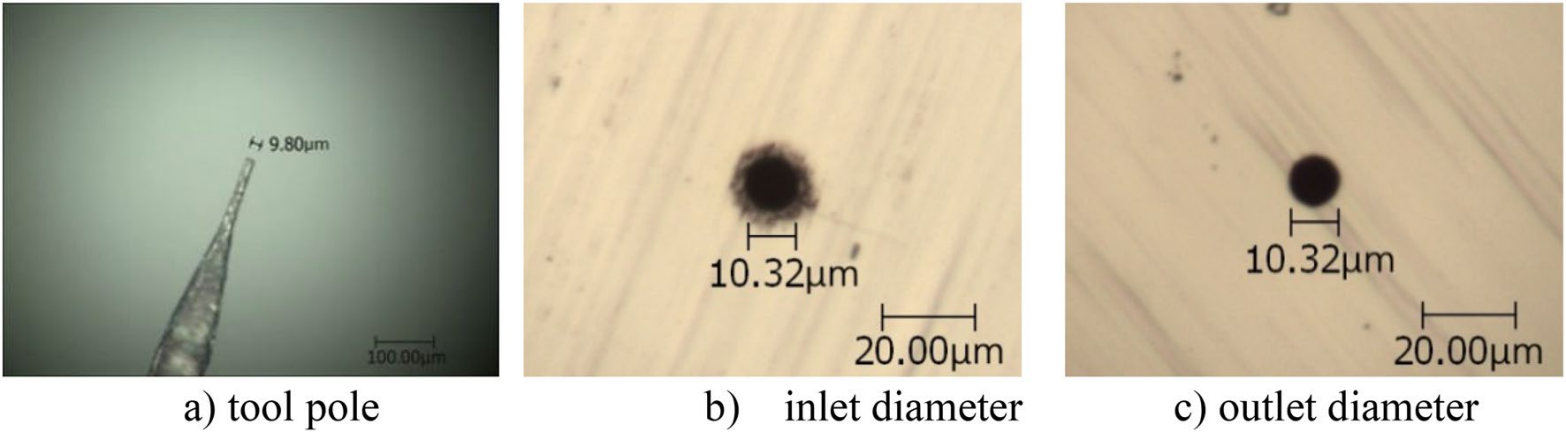
Micro holes produced at *k*_1_ = 0.92 and *k*_2_ = 0.

In the test, the diameter of the tungsten wire electrode used was 9.80 μm (see Fig. 1a). The frequency of pulse voltage was 100 kHz, and the duty cycle was 15%, which corresponded to 1.5 μs pulse width. The peak voltage was 1.3 V. Nickel sheet that was 5 μm thick was used as the anode work-piece, and the concentration of electrolyte dilute H_2_SO_4_ was 0.03 M. In the processing test of micro holes, the tool electrode is fed along the direction perpendicular to the workpiece, the feed depth is 8 μm, the feed speed is 0.03 μm/s, and the initial distance between tool and workpiece is 0.8 μm.

Under the same process parameters, the machining results of micro holes with different positive feedback gain *k*_1_ were obtained (see Fig. 1b and c, in which only one result is given due to space limitations). Changes in the average diameter of micro holes and the machining side gap, along with the positive feedback gain *k*_1_, are shown in Table 1. The machining resolution was estimated by the machining side gap, which was equal to half of the difference between the final hole diameter and the tool diameter.

**Table 1:** Micro holes produced for various *k*_1_.

*k*_1_	Average diameter(μm)	Side gap (μm)
0	31.42	10.8
0.4	23.50	6.85
0.6	18.53	4.37
0.8	13.59	1.90
0.92	11.02	0.61
0.93	14.24	2.22

Results show that in the case of positive feedback coefficient *k*_1_ < 0.92, with the increase of gain *k*_1_, the machining side gap decreases and the machining precision is improved. At *k*_1_ = 0.92, the average side gap reaches 0.61 μm. When k_1_ is above 0.92, the number of short-circuits in the processing significantly increases, and the repeated "feed-short-back" leads to the decrease of machining ability. The average side gap increases to 2.22 μm at *k*_1_ = 0.93. This is because the electrochemical reaction could not be carried out effectively. Thus, the positive feedback coefficient *k*_1_ should be taken to be below or equal to 0.92 in electrochemical micromachining with a positive feedback loop (Table 2).

**Table 2:** Micro holes produced for various *k*_2_.

*k*_2_	Average diameter(μm)	Side gap (μm)
0	29.95	10.30
0.4	22.10	6.37
0.6	18.72	4.68
0.8	14.31	2.48
1.0	12.30	1.48
1.2	9.92	0.29
1.3	15.04	2.85

### Effects of negative feedback gain on machining accuracy

**Figure 2:**
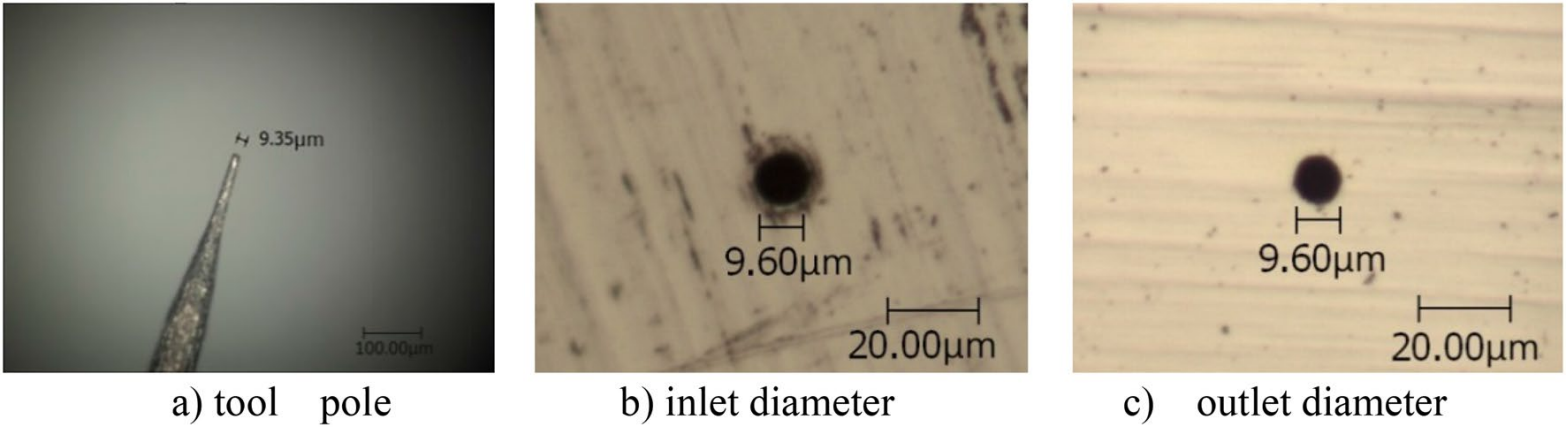
Micro holes produced for various *k*_1_ = 0 and *k*_2_ = 1.2.

From E's. (4) and (5) (see method section), it is known that increasing the negative feedback coefficient *k*_2_ can also extend the time constant A_v_ to improve machining accuracy. The verification was carried out through the micro-hole machining experiment, and each group of experiments was also repeated three times. Tungsten wire of 9.35 μm in diameter was used as the electrode in the experiment (see Fig. 2). Other parameters were taken to be the same as those of the positive feedback situation above.

Results show that with the increase of negative feedback gain *k*_2_, the machining side gap is decreased and the machining precision is improved. At *k*_2_ = 1.2, the average side gap reaches 0.29 μm. When *k*_2_ is above 1.2, the number of short-circuit in the processing significantly increases, and the repeated "feed-short-back" leads to the decrease of machining ability. The average side gap increases to 2.85 μm at *k*_2_ = 1.3. This is because the agrochemical reaction could not be carried out effectively. Thus, the negative feedback coefficient *k*_2_ should be taken to be below or equal to 1.2 in agrochemical micro machining with a negative feedback loop.

### Changes of machining accuracy along with gain ***k***_1_ for a given gain ***k***_2_

In chapter 2.1, test results show that feedback gain *k*_1_ should be at the range of 0–0.92. In chapter 2.2, test results show that feedback gain *k*_2_ should be at the range of 0–1.2. Here, we investigate effects of the feedback gain *k*_1_ on the machining accuracy for a given feedback gain *k*_2._ Here, the larger the feedback coefficient *k*_2_, the smaller the adjustment range of coefficient *k*_1_. In order to ensure that the coefficient *k*_1_ has a large adjustment range, the feedback coefficient *k*_2_ here takes a smaller number within the desirable range, and is taken as feedback gain *k*_2_ = 0.2.

**Figure 3:**
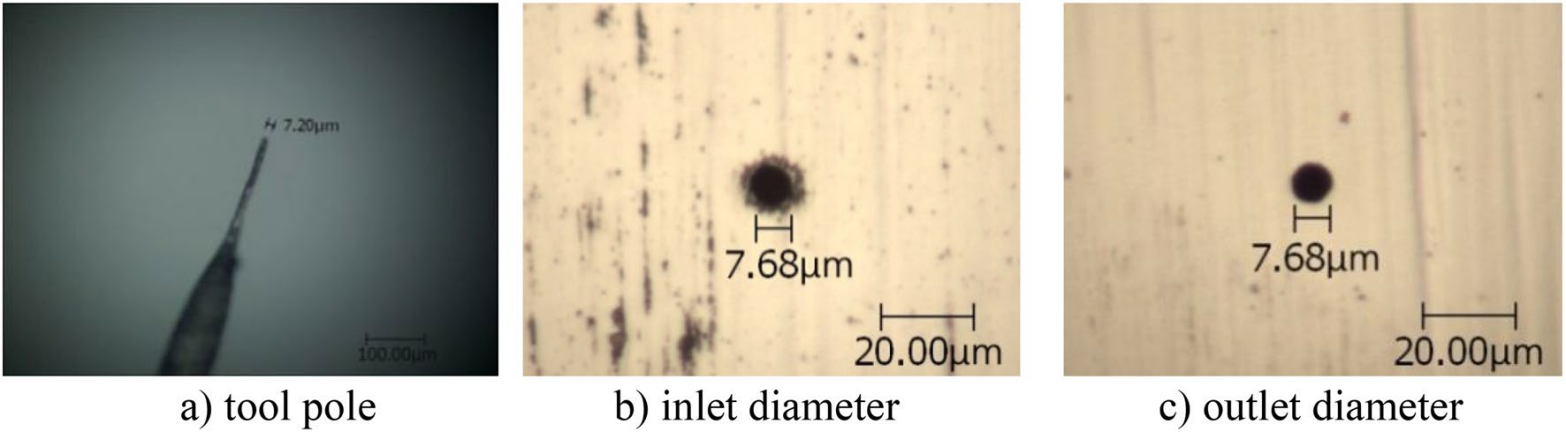
Micro holes produced at *k*_1_ = 0.64 and *k*_2_ = 0.2.

For a given negative feedback gain *k*_2_ = 0.2, the positive feedback gain *k*_1_ was changed to study the influence of the double feedback loop on the machining accuracy of micro holes by experiment, and each group of experiments was repeated three times. The tool electrode was tungsten wire of 7.20 μm in diameter (see Fig. 3a). Other parameters were taken to be the same as those in the two situations above.

Under the same process parameters (here *k*_2_ = 0.2), the machining results of micro holes with different positive feedback coefficient *k*_1_ were obtained (see Fig. 3b and c, in which only one typical result is given due to space limitations). Changes of the average diameter of micro holes and the machining side gap along with the positive feedback gain *k*_1_ are shown in Table 3.

**Table 3:** Micro holes produced for various *k*_1_ at *k*_2_ = 0.2.

*k*_1_	Average diameter(μm)	Side gap (μm)
0.2	21.70	5.98
0.4	15.60	2.93
0.6	10.81	0.54
0.64	7.59	0.20

Results show that at negative feedback gain *k*_2_ = 0.2, the machining side gap is still decreased and the machining precision is improved with increasing positive feedback gain *k*_1_. Compared with results for *k*_2_ = 0, the machining precision is improved more rapidly with increasing gain *k*_1_. At *k*_2_ = 0.2 and *k*_1_ = 0.64, the average side gap reaches 0.20 μm. It illustrates that the accuracy of the double feedback technology is higher than that by single positive feedback or single negative feedback technologies.

### Changes of machining accuracy along with gain ***k***_2_ for a given gain ***k***_1_

Here, we investigate effects of the feedback gain *k*_2_ on the machining accuracy for a given feedback gain *k*_1._ In a same manner as chapter 2.3, the larger the feedback coefficient *k*_1_, the smaller the adjustment range of coefficient *k*_2_. In order to ensure that the coefficient *k*_2_ has a large adjustment range, the feedback coefficient *k*_1_ here takes a smaller number within the desirable range, and is taken as feedback gain *k*_1_ = 0.2.

For a given positive feedback gain *k*_1_ = 0.2, the negative feedback gain *k*_2_ was changed to study the influence of the double feedback loop on the machining accuracy of micro holes by experiments, and each group of experiments was repeated three times.

**Figure 4:**
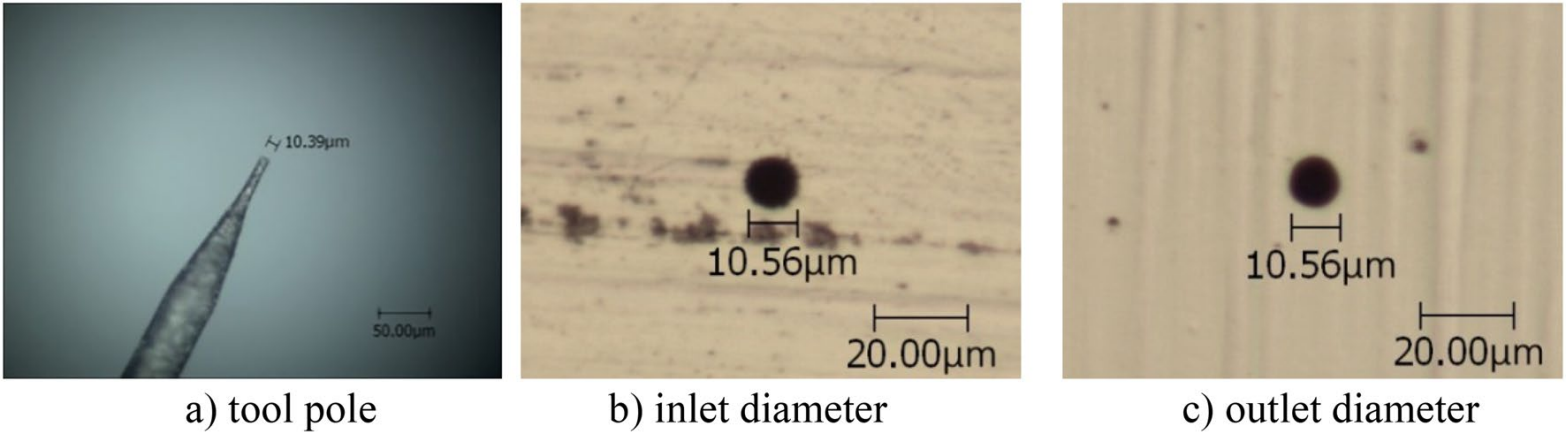
Micro hole produced at *k*_2_ = 0.7 and *k*_1_ = 0.2.

The diameter of the tool electrode was 10.39 μm (see Fig. 4a). Other parameters were taken to be the same as those in the situations above.

Under the same process parameters (here *k*_1_ = 0.2), the machining results of micro holes with different negative feedback coefficient *k*_2_ were obtained (see Fig. 4b and c, in which only one typical result is given due to space limitations). Changes of the average diameter of micro holes and the machining side gap, along with the positive feedback coefficient *k*_2_ are shown in Table 4.

**Table 4:** Micro holes produced for various *k*_2_ at *k*_1_ = 0.2.

*k*_2_	Average diameter(μm)	Side gap (μm)
0.2	22.10	6.18
0.4	15.07	3.96
0.6	12.12	2.49
0.7	10.81	0.21

Results show that at positive feedback gain *k*_1_ = 0.2, the machining side gap still decreases and the machining precision is improved with increasing negative feedback gain *k*_2_. Compared with results for *k*_1_ = 0, the machining precision is also improved more rapidly with increasing gain *k*_2_. At *k*_1_ = 0.2 and k_2_ = 0.7, the average side gap reaches 0.21 μm. This result further illustrates that the accuracy of the double feedback technology is higher than that by single positive feedback or single negative feedback technologies.

It should be noted that the values in Tables 1, 2, 3, 4 are different from those in Figs. 1, 2, 3, 4. This is because Tables 1, 2, 3, 4 show the average value of the multiple machining results, and Figs. 1, 2, 3, 4 give one case of multiple machining results.

Figures 1, 2, 3, 4 also show that the inlet and outlet diameters of the processed micro holes are basically the same as each other, and the taper of these micro holes is quite small.

**Figure 5:**
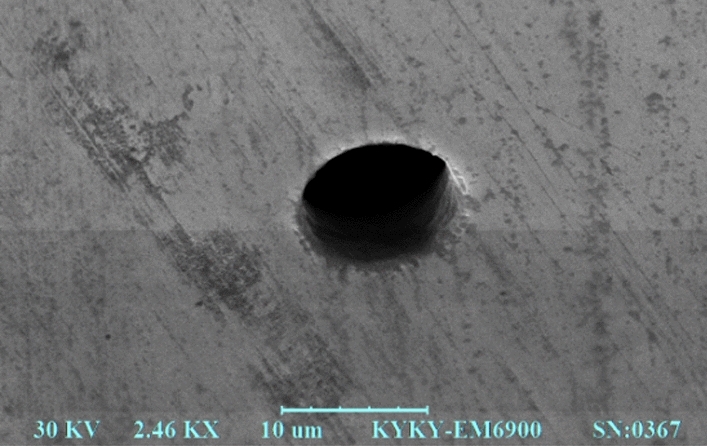
EM photo of micro hole at *k*_2_ = 0.7 and *k*_1_ = 0.2.

**Figure 6:**
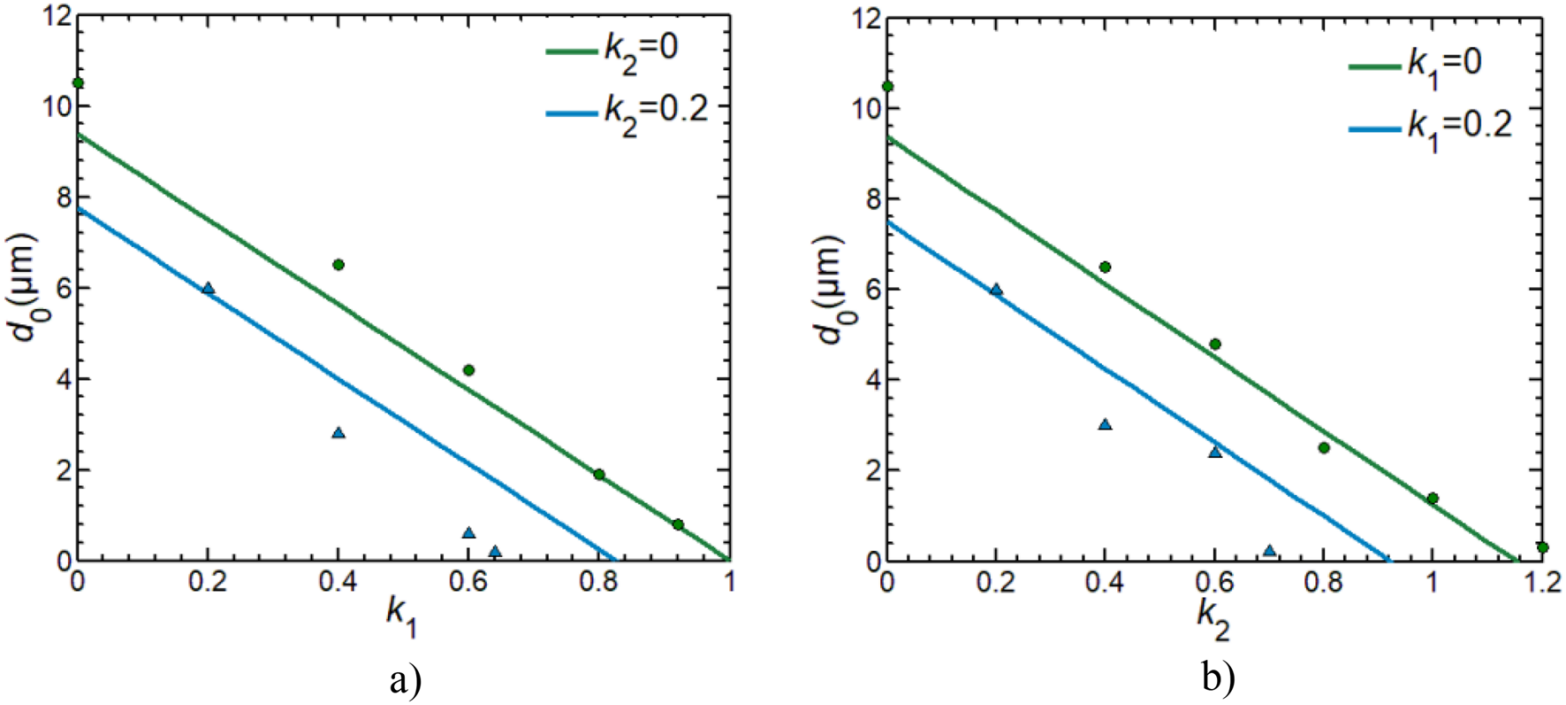
Changes of machining precision along with gains *k*_1_ and *k*_2_. lines: calculated *d*_0_. triangles and circles: tested *d*_0_.

**Figure 7:**
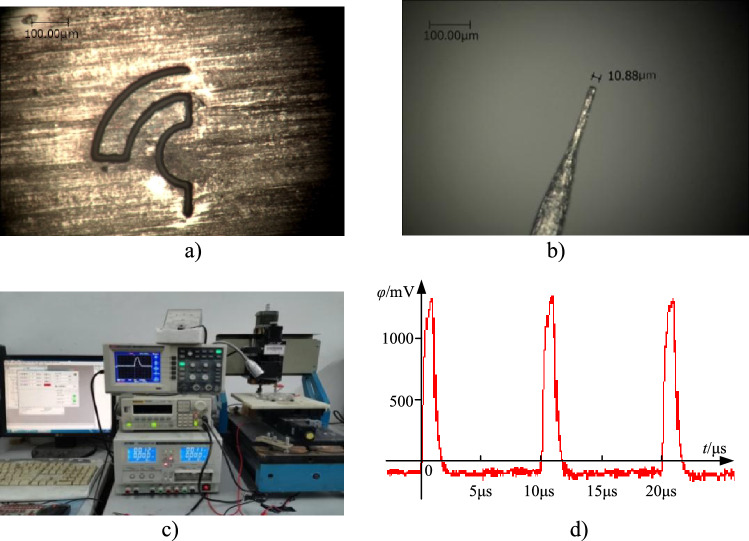
(**a**) Micro double curve beam produced with proposed technique. (**b**) Tool electrode; (**c**) machining system with double feedback circuits. (**d**) Experimental voltage waveform.

**Figure 8:**
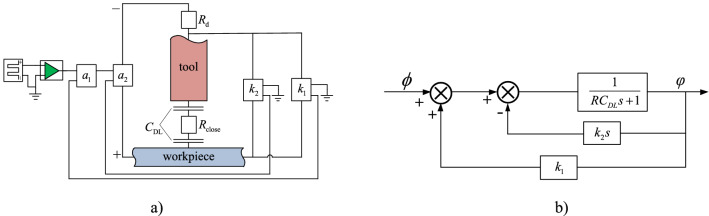
(**a**) Circuit of the machining system with double feedback loops; *a*_1_ and *a*_2_ denote adder; *k*_1_ is an proportional amplifier, *k*_2_ is an differential amplifier. (**b**) Block diagram of the equivalent circuit.

The experimental results show that the taper of the micro structures mainly depends on the taper of the tool electrode. Thus, the key is to ensure the cylindricity of the tool electrode. When the tool electrode tungsten wire is produced in the electrochemical principle, if the current is small, taper will occur on the tool electrode. There is an optimal current corresponding to the zero taper of the tool electrode. The micro holes in Figs. 5, 6, 7, 8 are produced by the tool poles under the optimized current.

Additionally, the tool electrodes used are only about 10 μm in diameter so that bending and other failures occur easily in the process of machining. After one tool electrode is damaged, one new tool electrode should be produced. The tool electrode of 10 μm diameter was formed by electrochemical etching of 100 μm tungsten wire in NaOH solution. Because the diameters are too small, the tool electrode diameter could not be exactly controlled, even under the same parameters. Considering that the accuracy of the micro holes is determined by the diameter difference between the tool electrode and the hole, several tool poles with similar but different diameters were used in the micro hole machining experiments, as shown in Figs. 1, 2, 3, 4.

An electron microscope (EM) picture is used to give surface topography of the micro hole, as shown in Fig. 5. The image is taken from an angle of about 45 degrees to see the hole wall. The EM picture shows the surface topography of the hole wall, illustrating that the machining surface quality of the micro hole is quite good.

### Comparison of test and simulation

Machining accuracy of the double feedback technique was simulated (see Fig. 6). Here, the machining accuracy as functions of the gains *k*_1_ and *k*_2_ is given. The simulation was performed using Eq. (5), and the simulated results were compared with experimental results. Here, *R*_e_ = 100 Ω cm (0.03 M H_2_SO_4_), C_d_ = 15 μF/cm^2^, and C_d_ is determined to be 15 μF/cm^2^. The experimental data was obtained from the above-mentioned tests, as shown in Tables 1, 2, 3, 4. Results show that:

When a pulse width *t*_on_ is given, the limit distance *d*_0_ is the maximum at *k*_1_= *k*_2_=0. Besides it, the limit distance *d*_0_ decreases linearly with increasing *k*_1_ for a given *k*_2_. At *k*_2_=0, the limit distance *d*_0_ approaches zero when *k*_1_ is near to 1. At *k*_2_=0.2, the limit distance *d*_0_ decreases more rapidly with increasing *k*_1_. At *k*_2_=0.2, the limit distance *d*_0_ approaches zero when *k*_1_ is near to 0.83.

In the same manner, the limit distance *d*_0_ decreases linearly with increasing *k*_2_ for a given *k*_1_. At *k*_1_=0, the limit distance *d*_0_ approaches zero when *k*_2_ is near to 1.15. At *k*_1_=0.2, the limit distance *d*_0_ also decreases more rapidly with increasing *k*_2_. At *k*_1_=0.2, the limit distance *d*_0_ approaches zero when *k*_2_ is near to 0.86.

The tested accuracy fits with the calculated one, illustrating the effectiveness of the proposed machining technology with double feedback circuits. These results mean that the machining accuracy of micro structures can get to nanometer scales by tuning the gain coefficients *k*_1_ and *k*_2_ under the condition that a pulse voltage with μs order of magnitude is used.

### Machining double micro curve beam

Using the machining method, one micro double cure beam was produced. Using the same electrolyte type and concentration as above, the micro double cure beam was also produced. The workpiece material was Ni. Here, *ϕ* = 1.3 V, *t*_on_ = 1.5 μs, *k*_1_ = 0.64, and *k*_2_ = 0.2. The main processing parameters were the same as the optimal parameters for machining micro holes. The only different parameter was the lateral tool feed rate. The optimal experiments were carried out, showing that the optimal tool feed rate was 0.09 μm/s.

Figure 7a shows a micro double curve beam machined using the technique with double feedback circuits. Here, the diameter of the tool electrode is 10.88 μm (see Fig. 7b).

Figure 7c shows the electrochemical micromachining system with double feedback circuits. It is mainly composed of a motion platform, control unit, power source, cathode electrode and anode workpiece fixture, and state detection elements. Figure 7d gives pulse voltage waveform after using double feedback circuit. It shows that, with the feedback loops, the voltage response time of the system to pulse signals increases, confirming that the time constant increases significantly due to the feedback signals.

To achieve submicron machining accuracy, the feed resolution of the motion platform needs to be maintained at the nanometer level. The micro-motions at the X, Y, and Z axes were driven with an 01TS001 ultra-thin electric translation platform with the initial resolution of 0.3125 μm. With 200 times of subdivision, the resolution could reach 12.5 nm. The corresponding macro motion in Z direction was driven by 01TS102 ordinary electric translation platform with a travel of 100 mm to ensure the operation space for clamping the anode workpiece and tool electrode.

The PCI-1240U 4-axis motion control card output pulses of 1 LSB precision, and the pulse range was 1PPS-4MPPS. With Active DAQ control of Visual Basic language, the library function of the motion control card was employed to write the motion control program. The motion speed of the micro-motion platform was controlled by setting the output pulse number per second. Machining time, motion distance, and short circuit number were displayed in real time.

The tool pole first etches vertically to 8 μm deep, through the workpiece, and then the tool moves laterally along a specified path in the Ni film as below: (a) The tool electrode travels in a straight line; (b) the tool electrode travels in a half-arc; (c) the tool electrode travels the second straight line; (d) the tool electrode travels the first 1/4 arc; (e) the tool electrode travels the third straight line; (f) the tool electrode travels the second 1/4 arc. The vertical and lateral tool feed rates are 0.03 μm/s and 0.09 μm/s, respectively.

The feeding motion for machining the double curve beam adopts the method of “discrete-interpolation”; that is, the points on the curve are firstly discretized, and then the interpolation feeding in X and Y directions is carried out.

For a given tool feed rate, a too-small discrete distance could make the tool electrode feed repeatedly in the interpolation process, causing a decline in machining precision. Too large discrete distance could cause slow retraction of the tool pole when a short circuit occurs. Thus, the discrete distance should be set properly. For 1/2 arc, its radius is relatively small (40 μm), and the corresponding discrete distance is taken to be 0.02 rad. For two 1/4 arcs, their radii are relatively large (80 and 120 μm), so the corresponding discrete distance is taken to be 0.01 rad.

The length of the machined micro double curve beam is approximately 180 and 120 μm, respectively. The trough width is about 12.46 μm. The distance from the tool to the trough wall is about 790 nm [(12.46 − 10.88)/2 = 0.79 μm]. The accuracy is much better than 2 μm given with 40 ns pulses^4,19^. Here, only 1.5 μs pulse width signals are used.

## Discussion

In this paper, an electrochemical micromachining method based on double feedback circuits was proposed. A positive feedback circuit plus a negative feedback circuit was used to control the micromachining system. The dynamic circuit equation of the system was then presented and used to determine machining accuracy as a function of the pulse width and feedback gains. The control circuit for the machining technique was designed and fabricated. Using the circuit, the effects of feedback loop gains on machining accuracy of the micro holes were studied. Experiments showed that all of the three feedback modes could increase machining accuracy. Among them, the double feedback mode had the highest machining accuracy. The tested accuracy was in good agreement with simulations. Using the technique, one micro double cure beam was produced, and its accuracy reached nanometer level under the condition of using an ordinary pulse duration power source.

When the stable voltage on DL layer is above the threshold voltage for electrochemical machining, the condition for effective machining under pulse voltage is that the pulse duration *t*_on_ is above the time constant τ_v_ (*t*_on_ ≥ τ_v_). Under the condition of *t*_on_ ≥ τ_v_, the closer the two are, the less effective machining time is for each pulse, and the higher machining accuracy is. As the distance from tool pole to the workpiece is small, the constant τ_v_ is small. Thus, to increase machining accuracy, small pulse duration *t*_on_ of the voltage signals is required.

In the proposed method, the small time constant τ_v_ at small distance between tool pole and workpiece can be tuned to be become a large value by tuning feedback gains. Therefore, the machining accuracy can get to nanometer lever using an ordinary pulse power source of μs pulse width. The double feedback circuits have higher tuning accuracy of the time constant τ_v_, which can make the time constant τ_v_ closer to pulse duration *t*_on_. Thus, the limit distance d_0_ is the smallest, and the machining accuracy is the highest with the double feedback circuits.

In electrochemical micromachining, the main method to improve the processing accuracy is to reduce the pulse width of the voltage signal, while the processing efficiency decreases. For example, in ref^7^, the pulse width is 50 ns, and the machining clearance reaches 2 μm. The feed speed for vertical drilling is only 0.06 μm/s. If the machining clearance is required to reach the nanometer level, the pulse width needs to be reduced to the picosecond level, and the machining feed speed needs to be further greatly reduced. In the proposed method, the pulse width is 1.5 μs. The machining clearance reaches 200 nm. The feed speed for vertical drilling is 0.03 μm/s. It is 1/2 of the feed speed in ref^7^ for 2 μm machining clearance. If compared under the same machining clearance, The feed speed of the proposed method should be much quicker than that of the method in ref^7^. Of course, compared with the ultra short pulse electrochemical micromachining technology, the advantage of our method is not only to improve the processing efficiency. The main advantage of our method is that using μs level pulse signals plus simple control circuit, the electrochemical machining accuracy is improved to nanometer level. It avoids the requirement of reducing the pulse width greatly in reference^7^, that is, it can avoid using the expensive ultra short pulse power supply.

If a resistance is connected in series in the electrochemical machining system, the micromachining accuracy could also be improved to a certain extent, but there are two shortcomings:

First, the series resistance will reduce the voltage applied between the two electrodes. In order to ensure the normal electrochemical machining, the output voltage of the pulse power supply must be increased. Second, the series resistance only can improve the time constant of the whole system composed of the resistance and the electrochemical machining unit, rather than the time constant of the electrolyte system, and the improvement of the machining accuracy will be limited.

The feedback control method proposed here can improve the time constant of the electrolyte system and is more effective for improving the machining accuracy. From Eq. (4), it can be known that increasing the feedback gain coefficient *k*_1_ can not only increase the time constant of the electrolyte system, but also increase the response voltage of the electric double layer. It means that the output voltage of the pulse power supply could be decreased. It is quite different from the case of series resistance.

## Methods

The pulsed electrochemical micromachining system is equivalent to an RC circuit, and its dynamic equation is^18^:

1 $$ {\mathbf{\varphi }} + {\varvec{SC}}_{{\mathbf{d}}} {\varvec{R}}_{{\varvec{e}}} \frac{{\user2{d\varphi }}}{{{\varvec{dt}}}} = \emptyset $$

where φ and *C*_d_ is the voltage and capacitance per unit area on double layer (DL) of the electrode, respectively; *S* is electrochemical reaction area on the electrode; *R*_e_ electrolyte resistance; *t* the time; $\emptyset$ the voltage applied to electrodes.

When double feedback loops are added, the circuit is changed (see Fig. 8a). Here, one negative feedback loop and one positive feedback loop are added.

The positive and negative feedback loops consist of a proportional amplifier and a differential amplifier, respectively. The voltage between the two poles is taken as the feedback signal. The feedback signal is applied to adder 1 through a reverse differential amplifier, and a negative feedback loop is formed. Meanwhile, the feedback signal is applied to adder 2 through a proportional amplifier, and a positive feedback loop is formed as well. Figure 8b provides a corresponding block diagram of the system.

The dynamic circuit equation of the machining system proposed is:

2 $$ \user2{\varphi } + {\varvec{SC}}_{{\varvec{d}}} {\varvec{R}}_{{\varvec{e}}} \frac{{\user2{d\varphi }}}{{{\varvec{dt}}}} = \emptyset - {\varvec{k}}_{2} {\varvec{CR}}\frac{{\user2{d\varphi }}}{{{\varvec{dt}}}} + {\varvec{k}}_{1} \user2{\varphi } $$

where *C* and *R* are the capacitance and resistance of the differential amplifier, respectively.

From Eq. (2), the corresponding control equation can be given as:

3 $$ \frac{\varphi \left( s \right)}{{\emptyset \left( s \right)}} = \frac{1}{{1 - k_{1} }}\frac{1}{{\frac{{k_{2} CR + R_{e} C_{DL} }}{{1 - k_{1} }}s + 1}} $$

Under unit step voltage, the voltage response on the double layer can be obtained based on Eq. (3) as:

4 $$ {{\varphi }}\left( {\text{t}} \right) = \frac{1}{{1 - k_{1} }}\left( {1 - e^{{ - t/\tau_{v} }} } \right) $$

Here, the equivalent time constant $\tau_{v} = \frac{{k_{2} CR + C_{DL} R_{e} }}{{1 - k_{1} }}$.

When the stable voltage on DL layer is above the threshold voltage for electrochemical machining, the condition for effective machining under pulse voltage is that the pulse duration *t*_on_ of the signals is larger than the time constant τ_v_ (*t*_on_ ≥ τ_v_). From it (considering *R*_e_ = *d ρ* S, *ρ* is the specific electrolyte resistivity and *d* is the distance between the tool pole and workpiece), the machining resolution as a function of the pulse duration *t*_on_, feedback gains *k*_1_ and *k*_2_ can be given as:

5 $$ d \le d_{0} = \frac{{t_{on} \left( {1 - k_{1} } \right) - k_{2} RC}}{{\rho C_{d} }} $$

where *d*_0_ is the limit distance in which the electrochemical reaction could occur, determining machining accuracy.

Equation (5) shows that the machining accuracy depends on the feedback loop gain *k*_1_ and *k*_2_, in addition to the pulse duration *t*_on_. From Eq. (4), we also know that the positive feedback loop increases the circuit voltage. To obtain the same machining voltage under different feedback gain levels, the electric source voltage should be adjusted according to the following condition:

6 $$ \emptyset_{v} = \emptyset (\left( {1 - k_{1} } \right)_{{}} $$

without a feedback loop, the most effective control parameter is the pulse duration *t*_on_. In the double feedback circuit method, feedback loop gains *k*_1_ and *k*_2_ can be used to reduce the limit distance *d*_0_.

**Figure 9:**
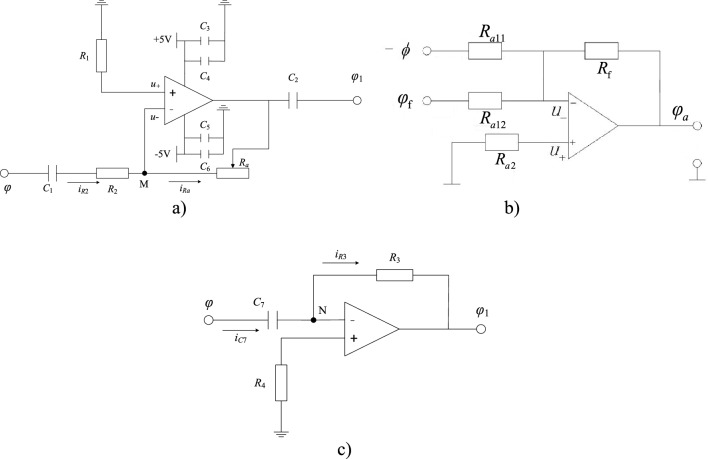
Feedback control elements. (**a**) Inverse proportional amplifier; (**b**) Inverse adder. (**c**) Inverse differential circuit.

In the feedback control circuits, the positive feedback circuit includes an inverse proportional amplifier (see Fig. 9a) and an inverse add circuit (Fig. 9b). In the inverse proportional amplifier, *R*_*a*_ is the feedback resistance, and is adjusted in the range of 0 to *R*_2_. The gain *k*_1_ of the inverse proportional amplifier is:

7 $$ k_{{1}} = R_{a} /R_{{2}} $$

Here, the adjusted range of the gain *k*_1_ is from 0 to 1.

In the inverse add circuit, *R*_*a*2_ is the balance resistance, *R*_f_ is the feedback resistance, and it is an adjusted resistance. Here, $\emptyset$ is the input voltage and *φ*_f_ is the feedback voltage. The output voltage of the adder is:

8 $$ \varphi_{a} = - (\emptyset + \varphi_{{\text{f}}} )R_{{\text{f}}} /R_{{{\text{a11}}}} $$

Here, *R*_a11_ is taken to be equal to *R*_a12_ (*R*_a11_ = *R*_a12_), and then the gain of the adder is *a* = *R*_f_/*R*_a11_*.* Taking *R*_f_ = *R*_a11_, then *φ*_*a*_ = -(∅ + *φ*_f_).

In the feedback control circuits, the negative feedback circuit includes an inverse proportional amplifier (see Fig. 9a), an inverse add circuit (Fig. 9b), and an inverse differential amplifier (see Fig. 9c). Here, the inverse adder and inverse proportional amplifier are the same as ones in the positive feedback loop. The gain of the inverse proportional amplifier is *k*_2_, which is also equal to *R*_*a*_/*R*_2_, and the adjusted range of the gain *k*_2_ is from 0 to 1 as well.

In the inverse differential circuit, *R*_3_ is the feedback resistance and C_7_ is the capacitance of the circuit. The output potential of differential circuit is:

9 $$ \varphi_{1} = - i_{R3} R_{3} = - R_{3} C_{7} \frac{d\varphi }{{dt}} $$

**Figure 10:**
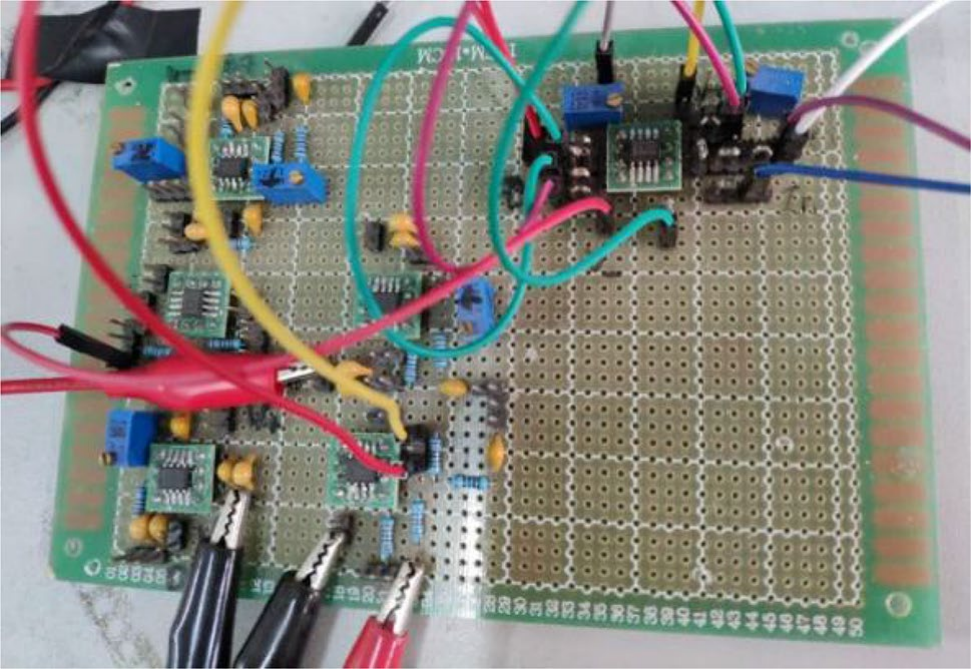
Photo of the feedback control circuits.

**Figure 11:**
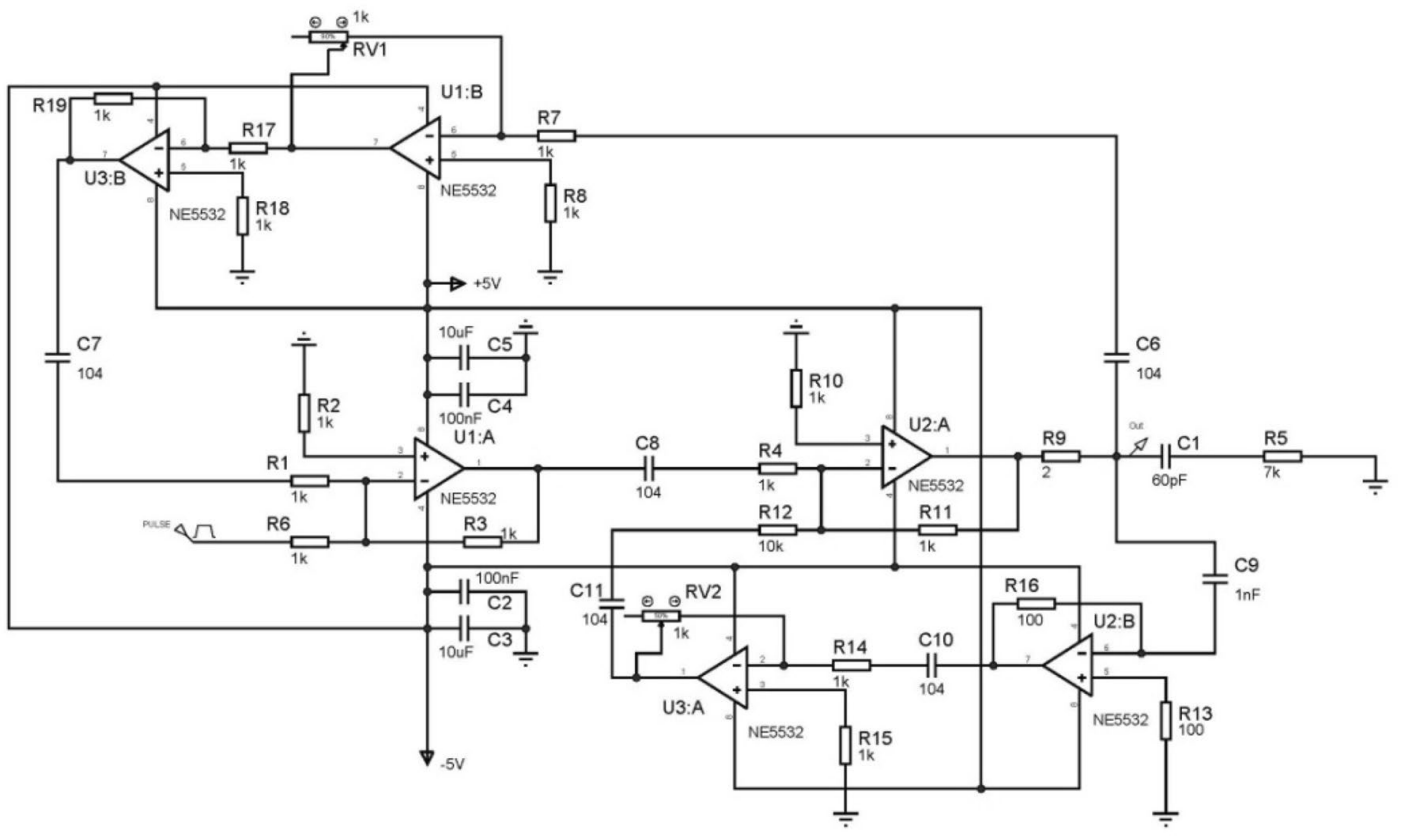
Control circuit for micro electrochemical machining with double feedback loops.

A photo of the feedback control circuits is given in Fig. 10. The circuit diagram of the entire system with double feedback control is given in Fig. 11.

## Data Availability

All identified data are available upon reasonable request from the corresponding author.
